# Supplementary material for: The CBS test: Development, evaluation & cross-validation of a community-based injury severity scoring system in Cameroon
Source: PLOS Glob Public Health. 2023 Jul 26;3(7):e0002110. doi: 10.1371/journal.pgph.0002110 (PMC10370767; doi:10.1371/journal.pgph.0002110)
Supplement: S1 Table — (DOCX) [file pgph.0002110.s002.docx]

**S1 Table 1. Frequency of CBS Indicators in Hospital-Based Injuries**

|  | **(+) CBS Indicator** | | **Association with Mortality** | |
| --- | --- | --- | --- | --- |
|  | *n* | *%* | *Odds Ratio* | *p* |
| CBS Indicator: |  |  |  |  |
| Stopped Breathing | 53 | 0.6% | 811.7 | <0.0001 |
| Unconscious | 408 | 4.3% | 29.1 | <0.0001 |
| Confused | 445 | 4.7% | 1.9 | 0.009 |
| Amnestic | 83 | 0.9% | 1.0 | 0.996 |
| Carried Off Scene | 5304 | 55.4% | 0.4 | <0.0001 |
