# Supplementary material for: The CBS test: Development, evaluation & cross-validation of a community-based injury severity scoring system in Cameroon
Source: PLOS Glob Public Health. 2023 Jul 26;3(7):e0002110. doi: 10.1371/journal.pgph.0002110 (PMC10370767; doi:10.1371/journal.pgph.0002110)
Supplement: Attachment — Submitted filename: Response to Reviewers on Letterhead.pdf [file pgph.0002110.s004.pdf]

UNIVERSITY OF CALIFORNIA, LOS ANGELES **UCLA**

BERKELEY • DAVIS • IRVINE • LOS ANGELES • MERCED • RIVERSIDE • SAN DIEGO • SAN FRANCISCO SANTA BARBARA • SANTA CRUZ

Dear Dr. Demetriades and reviewers,

Thank you for the opportunity to resubmit our revised paper “The CBS Test: Development, evaluation & cross-validation of a community-based injury severity scoring system in Cameroon” to PLOS Global Public Health. We appreciate the constructive and insightful comments of the reviewers and believe they have significantly strengthened the manuscript. We have detailed our responses below with the corresponding manuscript changes. We appreciate your time and ongoing consideration of our paper.

REVIEWER #1

• **Out of curiosity, why did you look at fuel sources? This was unclear in the manuscript.** Thank you for this question. Socioeconomic measures used in high-income countries with larger formal economies are often unreliable measures of socioeconomic status in low and middle-income countries with larger informal economies. Fuel type has commonly been used to construct socioeconomic indices in LMICs. Specifically, use of LPG fuel in Cameroon in rural areas is associated with a higher level of education, access to sanitation, piped water and household wealth (income and asset ownership), while use of LPG fuel in peri-urban areas is associated with increased access to sanitation, piped water and education (Citation 13: Pope et al, 2018). Consequently, we examined use of LPG fuel as a proxy for improved socioeconomic status. We have clarified this further in the manuscript in the results section (p.5, line 244-245).

REVIEWER #2

• **What does looking at the “presence or absence of CBS indicators” as a variable indicate with respect to the objective of this study? Please outline this connection explicitly. It wasn’t immediately clear that this is the variable of interest.** Thank you for this question. To summarize it here, an affirmative response to any of the CBS questions was considered to be a positive CBS test result (CBS+), potentially indicative of more severe injury. We have included an additional sentence in the last paragraph of the introduction for further clarity (p.2, lines 79-80).

• **Is the decrease in AUC acceptable to justify the use of CBS in this setting over other options? Under what conditions or justification? Please outline this to support your claim that the CBS test is a viable option for triage.** Thank you for this question. The adequacy of the AUC depends on the conditions of application (e.g. the consequences of misclassification, such as false negatives). An AUC of 0.8029 (sensitivity: 77.0%, specificity: 91.6%) represents good test discrimination and we find the slight decrease in mortality discrimination (AUC) compared to conventional scores to be acceptable when employed in the appropriate setting. Conventional injury severity scoring systems cannot be applied in the community setting. At present, there is no available test to triage patients in the absence of trained medical personnel and basic equipment, such as blood pressure monitors. The sensitivity of untrained, lay personnel in identifying severe injury is unknown. Compared to no triage test and random chance (AUC 0.5), an AUC of 0.8029 is sufficient. In summary, use of the CBS test is justified over conventional injury severity scoring systems when there is a lack of trained medical personnel and/or basic diagnostic medical equipment. We have included further information regarding this important point in the discussion section (p.11, lines 425-437).

• **Could you speculate on why the CBS+ and CBS- populations are different? What contributes to this? What would potential adopters of this scoring system need to think about?** Thank you for this question. While it is not possible to know etiology in cross-sectional data, we speculate that CBS+ and CBS- populations are different for that following reasons: With regards to clinical indicators (e.g. vital signs, intubation, transfusion, etc) and mortality, CBS+ populations are probably different than CBS- patients because they likely represent a group with 1) greater injury severity and 2) increased prevalence of head injury. With regards to demographic and socioeconomic variables, CBS+ patients are likely different than CBS- patients because of the risk factors for more severe injury, including male gender and poverty. Potential adopters need to be aware of the potential propensity of the CBS test to identify head injury, which we noted in the limitations section (p.11, lines 450-455). We have also included further discussion about differences between CBS+ and CBS- populations in the discussion with regards to injury severity and severe injury risk factors (p.10, lines 398-400). Finally, given that we cross-validated the CBS test in patients presenting to the hospital for formal evaluation (where the prevalence of more severe traumatic injury is higher than in the general population), CBS test adopters would need to continue to use sensitivity and specificity over false positive and false negative rates, as the former are not dependent on a condition's prevalence. Although we intentionally did not report false positive or false negative rates, we have also mentioned this as a caveat in the limitations section (p.11, lines 460-464).

• **What happened to trauma registry data collection after January 2020? Did something make data collection no longer possible? This would be helpful to outline if it gives context on feasibility of such data collection into the future. What needs to be considered here for something to work?** Thank you for this question. The Cameroon Trauma Registry continues to function robustly and has expanded to additional hospitals in Cameroon. After January 2020, the trauma registry criteria changed to only include patients who died or were admitted, transferred or discharged from the hospital. We have clarified this further in the manuscript to avoid confusion (p.4, lines 144-147).

• **You do more than simply cross-validate CBS here. You also look at (and make claims for) its feasibility of implementation. I would consider adding this point when you outline the study objective to set readers' expectations.** Thank you for this comment. We agree that this is a considerable strength of the data presented here. We have highlighted that informal assessment of the CBS test's feasibility in both the community and hospital setting was intrinsic to the study methodology (p.2, lines 87-88).

• **Given the number of potential injury severity scores, why do you think one of them hasn't been picked up and implemented regularly in the Cameroonian context? Similar efforts, like for KTS, have been carried out, and you suggest that even KTS is not appropriate here. In the introduction, it may be helpful to highlight issues specific to Cameroon that make existing approaches infeasible. For example, why does a community-based score work better than KTS, which was created for resource-constrained settings?** Thank you for this question. The utility of the injury severity score depends on the setting in which it is employed. We recommend the CBS test be used when there • are inadequate trained personnel and/or equipment to for other conventional tests. The CBS test also has the ability to be applied retrospectively, although this may be subject to recall bias. The difficulty calculating respiratory rate (a key component of KTS and RTS) may be more specific to the trauma setting than the resource-constrained setting. We have edited this section for enhanced clarity (p.10, lines 367-373, see also p. 11, lines 425-437).

Sincerely,

Haley Tupper

Haley Tupper, MD, MS, MPH
